# Memory Impairment, Pro-Inflammatory Host Response and Brain Histopathologic Severity in Rats Infected with *K. pneumoniae* or *P. aeruginosa* Meningitis

**DOI:** 10.3390/pathogens11080933

**Published:** 2022-08-18

**Authors:** Bassma H. Elwakil, Basant A. Bakr, Mohammed M. Aljeldah, Nourhan S. Shehata, Yahya H. Shahin, Zakia A. Olama, Maria Augustyniak, Mourad A. M. Aboul-Soud, Abeer El Wakil

**Affiliations:** 1 Department of Medical Laboratory Technology, Faculty of Applied Health Sciences Technology, Pharos University in Alexandria, Alexandria P.O. Box 21311, Egypt; 2 Department of Zoology, Faculty of Science, Alexandria University, Alexandria P.O. Box 21568, Egypt; 3 Department of Clinical Laboratory Sciences, College of Applied Medical Sciences, University of Hafr Al Batin, Hafr Al Batin 39524, Saudi Arabia; 4 Department of Botany and Microbiology, Faculty of Science, Alexandria University, Alexandria P.O. Box 21568, Egypt; 5 Faculty of Natural Sciences, Institute of Biology, Biotechnology and Environmental Protection, University of Silesia in Katowice, Bankowa 9, 40-007 Katowice, Poland; 6 Chair of Medical and Molecular Genetics Research, Department of Clinical Laboratory Sciences, College of Applied Medical Sciences, King Saud University, P.O. Box 10219, Riyadh 11433, Saudi Arabia; 7 Department of Biological and Geological Sciences, Faculty of Education, Alexandria University, Alexandria P.O. Box 21526, Egypt

**Keywords:** bacterial meningitis, brain pathology, dementia, bacterial infection, Alzheimer’s disease

## Abstract

Meningitis caused by *Klebsiella pneumoniae* and *Pseudomonas aeruginosa* has lately become a prevalent cause of the central nervous system (CNS) infection. Bacterial invasion into the subarachnoid space prompts the releasing mechanism of chemokines and pro-inflammatory cytokines. The present study aimed to compare *K. pneumoniae* and *P. aeruginosa* meningitis concerning the memory, pro-inflammatory mediators and brain histopathological changes at different time intervals in adult Albino rats. The animals were sacrificed at three time intervals comprising 5, 10 and 15 days after meningitis induction. Cerebrospinal fluid (CSF) culture, relative brain weights, complete blood analysis, biochemical markers, levels of cytokine, chemokine and brain-derived neurotrophic factor (BDNF), neurotransmitter acetylcholine esterase (AChE) activity, and the brain histopathology of the infected rats in comparison to those in the control group were assessed. There was a significant increase in the levels of pro-inflammatory cytokines and chemokines including TNF-α, IL-1β, IL-6 and AChE after 5 days of bacterial meningitis infection with both *K. pneumoniae* and *P. aeruginosa*. The histopathological analysis of the cerebral cortex in the *P. aeruginosa* meningitis model at different time intervals revealed abundant numbers of dilated and congested blood vessels with severe hemorrhage, cerebral infarct, intracellular and extracellular vacuoles, and gliosis. Fifteen days post infection, a significant reduction in the brain tissue weight was observed. The meningitis model employing *P. aeruginosa* exhibited more evident time-dependent severity compared to *K. pneumoniae*, which may advocate its validity as a simple and effective research model to study meningitis of the CNS. This model may be utilized for further investigation to ascertain the molecular and biological association between bacterial meningitis and the development of the pathophysiological hallmarks underlying Alzheimer’s disease in preclinical and clinical setups. Clinical extrapolation based on studies employing animal disease models should be carefully interpreted.

## 1. Introduction

Bacterial meningitis (BM) is considered one of the most serious infectious diseases of the central nervous system (CNS). BM poses a life-threatening global health problem, owing to its high fatality rates and associated long-term neurological sequelae [1,2]. It has been reported by the World Health Organization (WHO) that the worldwide rates of mortality and severe complications resulting from infection by bacterial meningitis are 10% and 20%, respectively [3].

Several complications have been associated with BM, including hematological (hemorrhages) and neurological disorders (e.g., seizures, cerebral infarction, sensory and motor deficit, impairments in both memory and learning abilities and hearing loss) [4,5,6]. The intensive scientific research interest focusing on Alzheimer’s disease (AD) failed to identify the main cause of its sporadic late onset because the β amyloid (Aβ) and tau protein levels remain normal [7]. Recently, a correlation between dementia and infectious diseases either through bacterial brain invasion or via disturbance of the host-immune response has been raised [7]. It was reported that the presence of infection throughout a 5 year follow-up period would increase the odds of developing AD [8]. It is well known that oral infections [9,10] and tooth loss [11] were associated with AD. In a study conducted by Ishida et al. [12], AD-like phenotypes in mice were produced as a result of *Porphyromonas gingivalis* meningitis. Moreover, *Chlamydia pneumoniae* [13], *Helicobacter pylori* [14] and fungi [15] were also documented as pathological agents in AD. Gram-negative bacteria (GNB) are a common cause of several diseases, e.g., gum/periodontal disease (*P. gingivalis*), pulmonary infections (*Klebsiella pneumoniae*, *Legionella*, *Pseudomonas aeruginosa*), gastroenteritis (*Escherichia coli*, *Shigella*, *Salmonella*, *Vibrio cholera*), urinary tract infections (*E. coli*, *Proteus mirabilis*, *Enterobacter cloacae*, *Serratia marcescens*, *Bacteroides*), sexually transmitted disease (*Neisseria gonorrhoeae*), ulcers (*Helicobacter pylori*) and meningitis(*Neisseria meningitidis*) [7]. Two main reasons attract the researchers to study the correlation between the Gram-negative bacterial infections and AD: first, the presence of an increased number of GNB in the human gut with age [16]; secondly, the GNB-induced periodontal disease has been repeatedly associated with AD [17,18]. *P. aeruginosa* and *K. pneumoniae* are frequent and devastating causative agents of nosocomial bloodstream infections, as healthcare-associated infections flourishing in hospital niches. In the case of patients with compromised immunity, the infection and its complications rapidly progress, leading to severe outcomes. The fatality rates of *P. aeruginosa* and *K. pneumoniae* leading to bacterial meningitis are still significantly high, despite the progress made in the medical-care settings; which are due, in part, to the rising incidence rates of hypervirulent and antibiotic-resistant strains [19,20]. An outbreak of bloodstream infections caused by these two bacterial strains has been recently reported at an outpatient chemotherapy center in Taiwan [21].

Nowadays, the use of animal experimental models, employing non-human primates (NHPs), is central to the investigation of human diseases. This is ascribed primarily to the fact that the stable animal models simulate, to a large extent, the common characteristics of human diseases, thereby enabling clinical researchers to investigate pathogenesis and identify effective preventative strategies to combat its development [22,23]. Therapeutic interventions to treat bacterial meningitis are mostly slow, owing to the acute nature of its onset as well as uncharacteristic symptomatic manifestations, thereby leading to high fatality rates, particularly in pediatric patients, and subsequent long-term neurological complications, including epilepsy and mental damage [24].

Both *K. pneumoniae* and *P. aeruginosa* are uncommon causes of meningitis. The pathogenesis processes leading to their capacity of invading the CNS are poorly elucidated. As an initial step to investigate the cause–effect relationship between AD and bacterial meningitis, the current study aimed to establish stable and reliable models of bacterial meningitis in NHPs, and to compare the severity of bacterial meningitis that is triggered by the two GNB, i.e., *K. pneumoniae* and *P. aeruginosa* meningitis, and its impact on memory impairment and levels of pro-inflammatory markers in the CNS of rats.

## 2. Material and Methods

### 2.1. Microorganisms

The *K. pneumoniae* and *P. aeruginosa* strains were clinically isolated from patients at the Al-Shatby Pediatric Hospital. These strains were isolated using the Vitek 2 automated system (BioMerieux, Craponne, France) at the Medical Research Center, Faculty of Medicine, Alexandria University [25].

### 2.2. Animal Model

A total of 35 eight week-old adult male Albino rats, weighing from 330 to 350 g, were employed in this study. The present experiment was performed in an approved animal care center in accordance with the Animal Care and Use Committee at the Faculty of Science, Alexandria University, and was in accordance with the International Standards for the Care and Use of Laboratory Animals of the European Community Directive of 1986; AU*/*07*/*20*/*04*/*19*/*2*/*02. The bacterial inoculations were performed under anesthesia consisting of an intraperitoneal injection with ketamine hydrochloride (100 mg*/*kg) and xylazine (10 mg*/*kg). The rats were divided into groups and underwent intranasal instillation, receiving either 200 µL sterile saline as a negative control (Sham, *n* = 5) or an equivalent volume of *K. pneumoniae* (*n* = 15) or *P. aeruginosa* (*n* = 15) suspension, one at a time. Forty-eight hours later, the meningitis was documented by a quantitative culture of 5 µL CSF obtained by puncturing the cisterna magna [26]. Five animals were sacrificed at each time interval, i.e., 5, 10, and 15 days after the meningitis induction.

### 2.3. Morris Water Maze Test

The animals’ learning and memory were tested by the Morris water maze [27]. The apparatus consisted of a circular water tank (160 cm diameter and 45 cm high) with a fixed rectangular platform (10 cm × 5 cm) below the water level (2 cm below). The rats training consisted of five sessions for 5 days with four trials in each session with a cutoff time of 120 s and 30 s trial interval. If the rats failed to escape through the platform, they were gently guided with a rod to the platform. The escape latency was recorded for each rat (the time spent to find the platform). The decrease in the latency time in relation to the first session was considered successful learning.

### 2.4. Blood Biochemical Tests

The potentiality of multiple organ failure was assessed by quantifying the levels of the various biochemical markers in the rats’ serum at each time interval. All of the parameters were measured in duplicate and the experiment was repeated twice. Each blood sample was centrifuged (20 min at 4000× *g*) to separate the blood serum. The liver function parameters, including alanine aminotransferase (ALT), aspartate aminotransferase (AST), total bilirubin, albumin, alkaline phosphatase and gamma-glutamyl transferase (GGT), the kidney function parameters comprising uric acid, creatinine and urea, total cholesterol, glucose, and the total protein tests were determined using an automated biochemical analyzer, (Mindray BS-200 (Tripura Medical System, Visakhapatnam, India).

### 2.5. Estimation of Hematological Parameters

The various hematological parameters, namely hemoglobin (Hb), red blood corpuscles (RBC), packed cell volume (PCV), mean corpuscular volume (MCV), mean corpuscular hemoglobin (MCH), mean corpuscular hemoglobin concentration (MCHC), platelets, total leukocyte count and differential leukocyte count were analyzed using a standard auto blood-analyzer, (Biobase Bk-6310, (Biobase, Jinan, China). All of the parameters were measured at each time interval in duplicate and the experiment was repeated twice.

### 2.6. Brain Collection, Histological Processing, and Homogenization

At selected time intervals after the meningitis induction (5, 10 and 15 days), the rats were sacrificed, and the whole brain of each rat was excised, cleaned, dried and then weighed. The relative brain weight was estimated by dividing the whole brain weight by the final body weight of each rat. A portion of each collected brain was immediately washed with normal saline, fixed with 10% formalin, and then dehydrated using ethanol. The tissue samples were embedded in paraffin, cut into 5 µm thick sections and then deparaffinized and stained with Ehrlich’s hematoxylin and eosin (H&E) stain for histological analysis. Another portion of the whole brain was carefully excised, then the cortical and hippocampal brain tissues were isolated. Parts of the whole brain, as well as individual brain tissue samples, namely the cortex and hippocampus, were minced and homogenized separately in a phosphate buffer (10 mM tris-buffer, pH 7.4) containing protease inhibitors in a Potter-Elvehjem-type homogenizer. Each homogenate was centrifuged at 10,000× *g* for 20 min at 4 °C to pellet the cell debris, and the supernatant was collected and preserved at −80 °C for further biochemical assays.

### 2.7. Assays of TNF-α, IL-1β, and IL-6 Concentrations

The pro-inflammatory markers (TNF-α, IL-1β and IL-6) were quantified in the brain according to the ELISA kit method described in Immunotag™ ELISA Kits. The absorbance was measured at optical density (O.D.) 450 nm in a microtiter plate reader (MicroLAB, Coimbatore, India).

### 2.8. Neurotransmitter Markers

The acetylcholine esterase (AChE) activity was measured in the supernatant of the infected rats’ brain homogenate, according to Ellman et al. [28]. The absorbance was measured at 412 nm.

### 2.9. Western Blot

The brain homogenate was mixed with a radioimmunoprecipitation buffer (Tris-HCl; pH 7.4), 150 mM NaCl, 1 mM EDTA, 1% Triton X-100, 0.1% SDS and 0.1% protease inhibitor cocktail). The mixture was centrifuged and then 50 μg of the denatured protein was mixed with loading buffer followed by separation on 12% SDS-PAGE gel. A nitrocellulose membrane was used to transfer the protein samples. The membrane was incubated in a blocking buffer (5% non-fat milk/PBS) for one hour. Then, the membrane was incubated with a primary antibody (BDNF, Novus Biologicals, Littleton, CO, USA; diluted 1:500) followed by a secondary antibody (horseradish-peroxidase, Santa Cruz, Dallas, TX, USA; diluted 1:1000). The immunoreactive bands were detected and quantified using UVIBAND Image software. The β-actin was used as an internal control.

### 2.10. Statistical Analyses

The data obtained were presented as the mean ± standard deviation (SD). The comparisons were performed using one-way analysis of variance (ANOVA) followed by post-hoc analysis Tukey’s HSD (honestly significant difference) test (*p* ≤ 0.05). The same letters in the Figures represent the homogeneous groups. The blood biochemical parameters are shown on heatmaps to compare a large number of data among the experimental groups and time points. All of the analyses were performed using the Statistica 13.3 software.

## 3. Results

### 3.1. Bacterial Count

**Figure 1:**
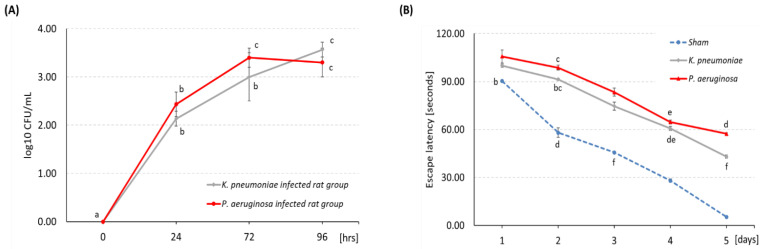
(**A**) The bacterial count in CSF samples to confirm the meningitis induction. It was established with 20 × 10^4^ CFU/mL; (**B**) The escape latency time over days (the same letter denote no significant difference among experimental groups tested within each time interval; ANOVA, Tukey test; *p* < 0.05).

After 24 h of *P. aeruginosa* and *K. pneumoniae* (5 × 10^5^ CFU/mL) inoculation, the CSF samples were used to confirm the meningitis induction. The bacterial count was relatively low (less than 300 CFU/mL of CSF), and it was established with 20 × 10^4^ CFU/mL (Figure 1A). There was no significant difference in the count of bacterial meningitis between the two tested bacteria (*p* ≥ 0.05).

### 3.2. Morris Water Maze Test

The escape latency was the required time for the rats to find the platform over 5 consecutive days. The average escape latency time was significantly prolonged in the bacterial meningitis groups compared to the control group (*p* < 0.05) (Figure 1B).

### 3.3. Effects of Bacterial Infection upon Blood Biochemical and Hematological Parameters

**Figure 2:**
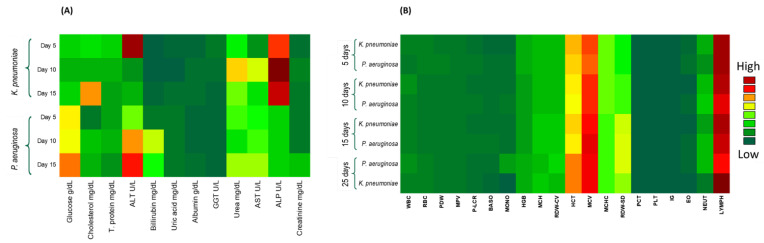
Heatmaps illustrating data on (**A**) Blood biochemical, and (**B**) Hematological parameters among experimental groups and time points. The scale shows the intensity of each parameter in unified units (green: lowest values; red: highest values).

In the present work, different biochemical parameters were evaluated in complementarity with recent studies that focused on the mortality causes in bacterial meningitis. The bacterial meningitis was associated with systemic complications (Figure 2A; Table 1). The cholesterol and glucose levels were significantly higher in both of the infected groups throughout the experimental period.

In the present study, the WBCs count in the *P. aeruginosa*-infected rat group steadily increased over time (Figure 2B; Table 2). However, the*K. pneumoniae*-infected rat group showed a slight fluctuation in their WBCs count through the experimental period. The neutrophils, lymphocytes, monocytes, eosinophils and basophils percentages were significantly high in all of the tested groups.

### 3.4. Measurement of Relative Brain Weight

**Figure 3:**
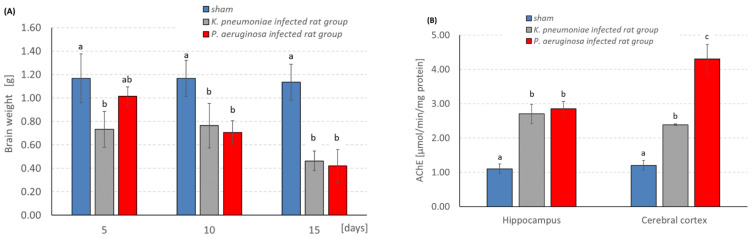
(**A**) The relative brain weight, and (**B**) The acetylcholine esterase (AChE) activity in the experimental groups (the same letters denote homogenous groups; ANOVA; Tukey test; *p* < 0.05).

The measurement of the relative brain weight was assessed during the experimental period and it was revealed that on day 5, the *P. aeruginosa*-infected rat group showed a significantly higher brain weight which was decreased by time and reached 0.47 g/rat weight on day 15 (Figure 3A). However, the *K. pneumonia*-infected rat group showed a steady decrease in brain weight as a function of time.

### 3.5. Effects of Bacterial Infection upon Brain Functional Activity

The assessment of the AChE activity after 15 days of bacterial meningitis infection in rats revealed a significant increase in AChE-specific activity in both the hippocampus and the cortex of the infected groups (Figure 3B). Interestingly, *P. aeruginosa* infection significantly increased the AChE activity in the cerebral cortex four times compared to the control group.

**Figure 4:**
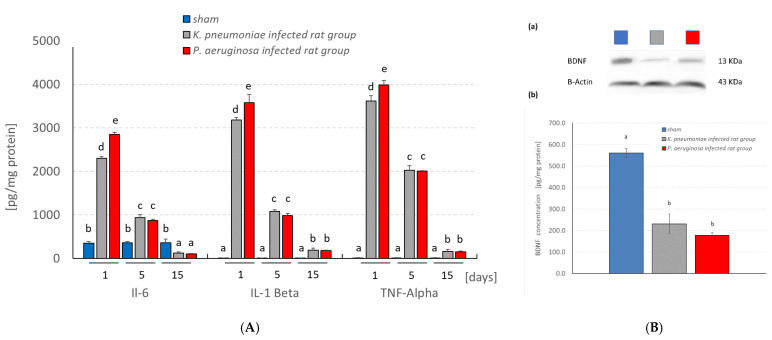
(**A**) Assays of TNF-a, IL-1β, and IL-6 concentrations in the experimental groups; and (**B**) Representative (**a**) Western blotting of BDNF protein, and (**b**) histogram showing BDNF concentration in the hippocampus of infected rats (the same letters denote homogenous groups within a given parameter; ANOVA, Tukey test; *p* < 0.05).

The ELISA quantification results (pg/mg protein) showed that intranasal administration of bacterial meningitis in rats significantly stimulated the pro-inflammatory cytokines production, TNF-α, IL-6 and IL-1β, compared with the controls (Figure 4A). The quantification of BDNF in the hippocampus homogenate of the sham and the meningitis rat models showed a significant decrease in the concentration in the infected rats compared to the sham ones (Figure 4B). However, no significant difference was noticed between the *K. pneumoniae*- and *P. aeruginosa*-infected rat groups.

### 3.6. Effects of Bacterial Infection upon Individual Brain Tissues Histoarchitecture

**Figure 5:**
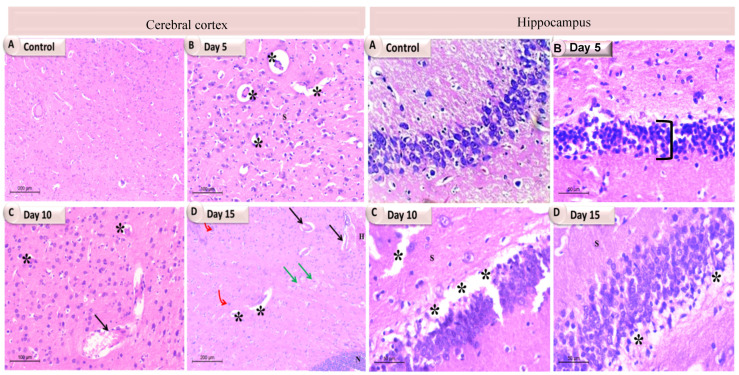
A photomicrograph showing the individual brain tissues at different time intervals of *K. pneumoniae* infected rats. In the cerebral cortex, (**A**) The control group showing normal cytoarchitecture with normal neurons; (**B**) Day 5 showing vacuolations (stars); (**C**) Day 10 showing dilated congested blood vessel (arrow) with perivascular edema and vacuolations (stars); (**D**) Day 15 showing dilated blood vessels (black arrows), severe congestion (green arrows), vacuolations (stars) and gliosis (red bent arrows), a large area of necrotic foci in the brain parenchyma along with lymphocyte infiltration and the presence of degenerating and/or apoptotic neurons (N). While in the hippocampus: (**A**) The control group showing normal cytoarchitecture; (**B**) Day 5 showing decreased thickness of the pyramidal layer (bracket); (**C**) Day 10 showing degeneration and vacuolation (stars); (**D**) Day 15 exhibited a number of vacuolations (stars).

**Figure 6:**
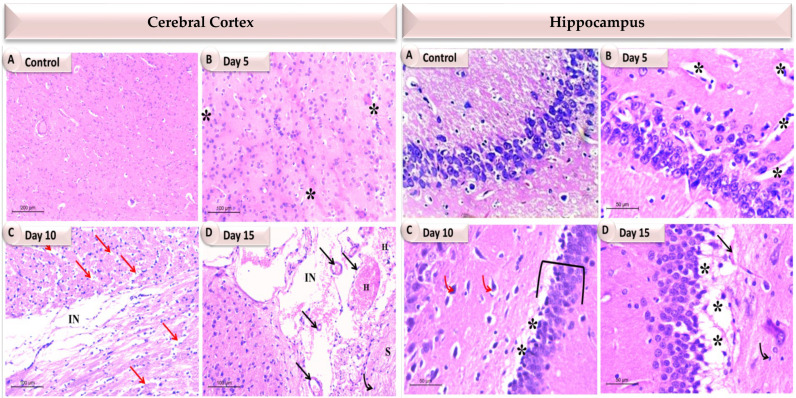
A photomicrograph showing the individual brain tissues at different time intervals of *P. aeruginosa* infected rats. In the cerebral cortex, (**A**) The control group showing normal neurons cytoarchitecture; (**B**) Day 5 showing more or less normal neuronal architecture with the appearance of a small number of vacuoles (stars); (**C**) Day 10 showing intracellular and extracellular vacuoles (red arrows), cerebral infarct (IN); (**D**) Day 15 showing an abundant number of dilated and congested blood vessels (black arrows) with severe hemorrhage (H), cerebral infarct (IN), gliosis (black bent arrows), spongy changes (S). While in the hippocampus, (**A**) The control group showing normal cytoarchitecture; (**B**) Day 5 showing scattered vacuoles (stars); (**C**) Day 10 showing a huge vacuolated layer separating the hippocampus layers (stars), a number of apoptotic neurons (red bent arrows), decreased thickness of pyramidal cell layer (bracket); (**D**) Day 15 showing dilated congested blood vessel ( black arrow), increased apoptotic neurons with dystrophic changes in the form of shrunken hyperchromatic, irregular with chromatolysis and abnormal, Nissl granule distribution (black bent arrow), abundant vacuoles (stars).

The pathological alterations in the cerebrum and hippocampus of the experimental rats were examined under light microscopy at different time intervals (5, 10 and 15 days) post infection. The H&E staining results reported obvious damage in the cerebral cortex, which varies with each time interval (Figure 5 and Figure 6; Figure S1, Supplementary Materials).

The cerebral examination of the 5th day group in both the *K. pneumoniae*- and the *P. aeruginosa*-infected rats showed a few vacuoles, in addition to a dilated congested blood vessel with perivascular edema in the *K. pneumonia* group. In addition, the cerebral cortex exhibited normal neuronal architecture with central large vesicular nuclei, containing one or more nucleoli, and peripheral distribution of the Nissl granules, reflecting the lower degree of damage at this time interval. Compared to the previous stage, the 10th day post-infection results revealed more damage manifested by a large number of internal and external vacuoles with an aberrant neuronal structure scattered in the spongy fibers in the *K. pneumoniae* group and large cerebral infarcts in the *P. aeruginosa* group. Furthermore, at the 15th day interval, which was the last one examined, extreme damage was exhibited in the *K. pneumoniae*-infected rats confirmed by severe vascular congestion, vacuolations and severe cellular neurofibrillary of the neuropil infiltration, along with a large area of necrotic foci in the brain parenchyma. Whereas, in the *P. aeruginosa* group, an abundant number of dilated and congested blood vessels with severe hemorrhage, the appearance of cerebral infarct, intracellular and extracellular vacuoles and gliosis were observed.

Moreover, histopathological changes in the hippocampus were observed simultaneously in both of the infected groups, as mentioned above (Figure 5 and Figure 6; Figure S2, Supplementary Materials). The 5th and 10th day experimental groups revealed mild to moderate damage, respectively. In the *K. pneumoniae* group, the appearance of vacuolation, tissue fibrillation and a few abnormal neurons structure were reported, while in the *P. aeruginosa* group, the 5th day group exhibited standard hippocampal architecture, reflecting less damage at this time interval. The 10th day group showed the appearance of a huge vacuolated layer separating the layers of the hippocampus, dilated blood vessels and a number of apoptotic neurons. In comparison to the sham rats and previous intervals, the 15th day animals showed thinning of the pyramidal layer, degeneration of pyramidal neurons and many vacuoles in the *K. pneumoniae* group, whereas in the *P. aeruginosa* group, the 15th day animals showed a decrease in the thickness of the pyramidal cell layer, a large number of apoptotic neurons with dystrophic changes in the form of shrunken hyperchromatic nuclei, irregular with chromatolysis and abnormal Nissl granule distribution. Many of the vacuoles were also evident.

## 4. Discussion

In the present work, meningitis rat models were designed to assess the *K. pneumoniae* and *P. aeruginosa* potentialities to induce memory impairment, increase the pro-inflammatory host response and alter the brain histoarchitecture. The cognitive decline during bacterial meningitis could be attributed to the elevation of the inflammatory mediators in the hippocampus and frontal cortex, which can damage cognitive function [29]. Hence, the impaired memory performance in both of the infected rat groups can be explained by several indications, e.g., the induction of cholinergic function by scopolamine which was reflected by the increasing activity of AChE in hippocampal and cortical regions. Sardari et al. [30] reported that the impairment in memory performance after intraperitoneal injection of lipopolysaccharides (LPS) was attributed to AChE hyperactivation, which led to nerve impulse cessation [31]. AChE plays a crucial role in AD by increasing the Aβ accumulation and stimulating the Aβ plaque formation and neurofibrillary tangles [32]. LPS can induce the inflammatory responses (including TNF-α stimulation from activated microglia) [33] and cell oxidative stress [34], which eventually leads to severe brain damage [35]. Barichello et al. [36] studied the memory impairment caused by *K. pneumoniae* meningitis and his study revealed that the TNF-α, IL-1b and IL-6 levels were elevated in the hippocampus during the experimental period. Other studies illustrated that the cytokines, namely IL-1b, IL-6 and TNF-α, can lead to dysregulation of several growth factors, including macrophage migration inhibitory factor, BDNF, fibroblast growth factor-2 and erythropoietin [36]. This was further explained through the in vivo administration of the pro-inflammatory mediators, which showed the ability to alter the cytoskeleton structure of endothelial cells that leads to blood–brain barrier paracellular dysfunction [37]. In the preclinical studies, researchers used LPS as a simple model for the induction of neurodegenerative changes encompassing the deposition of Aβ protein, cholinergic dysfunction and neuroinflammation that lead to sporadic AD [38]. In the present work, we used bacterial meningitis as a simple model for inducing neurodegenerative changes that trigger AD.

Our results illustrated the significant decrease in the BDNF concentration in *K. pneumoniae* and *P. aeruginosa* meningitis by using Western blot analysis. In addition, Barichello et al. [26] revealed that the *K. pneumoniae* meningitis induced the reduction in the BDNF levels in the hippocampus after 96 h post infection. On the other hand, Beheshti et al. [39] stated that the serum BDNF levels were notably elevated in patients with bacterial meningitis. In the same study, the BDNF levels in the CSF were linked to CSF blood platelet counts, interleukin IL-6 levels and neurological prognoses. BDNF neurotrophin is expressed in the T cells, B cells, activated monocytes, macrophages and neurons [40]. Angelucci et al. [41] revealed that the significant depletion of the pro-BDNF and mature BDNF levels in AD patients was positively correlated with AD’s degree of cognitive impairment. It was concluded that the BDNF levels in the brain could be a biological marker of preclinical AD.

In the present study, different biochemical parameters were assessed, and it was noticed that cholesterol and glucose were significantly higher in the *K. pneumoniae*- and *P. aeruginosa*-infected rats, respectively. It was discussed that the LPS induces AD-like cerebral changes, which were associated with other toxic effects in other organs, such as the kidney and liver. The hepatotoxic effect usually resulted from a significant increase in AST and ALT activity, with a significant reduction in the albumin level [42]. Sharew et al. [43] evaluated the mortality causes of bacterial meningitis in adults. The complications ranged from brain edema and vascular injury to circulatory and respiratory failure. The systemic complications (microbiological findings, blood biochemistry and brain pathology) can increase the mortality rate in meningitis patients. The abnormalities in insulin metabolism were among some of the major factors thought to influence the onset of AD. These abnormalities had a role in AD via influencing the synthesis/degradation of Aβ, and as a result of the neuronal alterations, danger/alarm signals from the oligomeric amyloid species would be provoked [44]. The alteration in the neuronal lipoproteins’ activity, in addition to cholesterol and other lipids, played a major role in the pathogenesis of neurodegenerative disorders [45]. Hypercholesterolemia was correlated to elevated levels of Aβ and linked with an increased AD risk. The cholesterol concentration can influence the metabolism of the amyloid precursor protein (APP) enzyme, in parallel with the Aβ production. However, those mechanisms are not fully understood [45]. Glucose homeostasis is also essential for neuronal maintenance, neurogenesis, neurotransmitter regulation, energy generation, cell survival, cognitive function and synaptic plasticity [44]. Glucose uptake/metabolism impairment also reduced the formation of acetyl-CoA [46], that may lead to an increased AD risk.

We studied several hematological parameters during the experimental period, and it was revealed that the neutrophils, lymphocytes, monocytes, eosinophils and basophils percentages were significantly high in both of the *K. pneumoniae* and *P. aeruginosa* meningitis rat models. In a previous study, bacterial meningitis diagnostic tests were assessed and it was stated that the number of WBCs, lymphocytes, neutrophils, eosinophils and monocytes percentages in all of the groups were significantly different [47]. They noticed that after the meningitis induction, the number of WBCs, eosinophils, neutrophils and monocytes significantly increased and the concentrations of potassium, glucose, AST and CK activity were also significantly increased.

*K. pneumoniae* and *P. aeruginosa* meningitis were studied in the present work. The histopathological architecture was investigated to compare the different effects of the two tested strains. It was revealed that the *P. aeruginosa* meningitis showed an increased severity in the cerebral cortex region with prolonged time, causing intracellular and extracellular vacuoles, cerebral infarct and an abundant number of congested blood vessels with severe hemorrhage, gliosis and spongy cytoarchitecture. Previously, in vivo and in vitro studies of the *K. pneumoniae* meningitis revealed that the main memory impairment mechanism was due to IL-17 induction due to TLR4 signaling. The IL-17 induction would influence the upregulation of granulopoietic cytokines involved in the recruitment of neutrophils [48]. On the other hand, *P. aeruginosa* induces the strongest inflammatory response, which can lead to septic shock. This common inflammatory response is attributed to the elaboration of certain extracellular toxins and enzymes, alkaline protease, *P. aeruginosa* elastase, exotoxin S, exotoxin A and phospholipase C [49,50]. This may explain the severity of *P. aeruginosa* meningitis in inducing memory impairment, increasing the pro-inflammatory host response and the observed histopathological alterations in the cerebral cortex and hippocampus. Furthermore, as the *P. aeruginosa*-induced meningitis model develops too fast and is more severe, this means that there is a narrow therapeutic drug window, which is life-threatening and may lead to serious therapeutic failures and/or adverse drug reactions. Therefore, therapy-related investigation is greatly required, together with drugs monitored closely for any signs of toxicity.

## 5. Conclusions

*K. pneumoniae* and *P. aeruginosa* are common cause of nosocomial infections but not of meningitis. The pathogenetic processes leading to their capacity for invading the CNS are poorly elucidated. As a result of our observations, we believe that the meningitis model using *P. aeruginosa* was more severe over time compared to *K. pneumoniae*. Herein, *K. pneumoniae* may be a good, simpler, and effective research tool to study the biological mechanisms involved in the pathophysiology of AD illness, including the induced memory impairment and the increased pro-inflammatory host response. To sum up, these models are of paramount significance for the identification of etiological factors leading to brain injuries, and for effective therapeutic strategies.

## Figures and Tables

**Table 1:** Mean ± SD of blood biochemical parameters in rats infected by *K. pneumoniae* or *P. aeruginosa* measured at various time points after infection (the same letters denote homogenous groups within a given parameter; ANOVA, Tukey test; *p* < 0.05).

Biochemical Parameters	*K. pneumoniae* Infected Rats (Mean ± SD)						*P. aeruginosa* Infected Rats (Mean ± SD)					
	Day 5		Day 10		Day 15		Day 5		Day 10		Day 15	
Glucose g/dL	100.00 ± 2.59	b	84.00 ± 5.20	a	111.77 ± 6.86	c	206.57 ± 6.65	d	207.65 ± 2.42	d	260.13 ± 8.54	e
Bilirubin mg/dL	0.21 ± 0.02	ab	0.14 ± 0.01	a	0.31 ± 0.05	b	0.58 ± 0.08	c	1.86 ± 0.13	e	1.43 ± 0.10	d
Uric acid mg/dL	2.65 ± 0.30	b	1.92 ± 0.08	a	3.14 ± 0.08	c	4.86 ± 0.23	d	4.78 ± 0.18	d	4.89 ± 0.19	d
Cholesterol mg/dL	117.81 ± 1.65	c	85.95 ± 2.81	b	258.73 ± 26.6	d	49.94 ± 0.95	a	97.33 ± 2.31	b	89.67 ± 6.35	b
Albumin g/dL	2.94 ± 0.08	ab	3.11 ± 0.02	b	3.38 ± 0.14	b	2.79 ± 0.16	a	2.95 ± 0.25	ab	3.69 ± 0.16	c
T. protein mg/dL	10.19 ± 0.01	e	8.49 ± 0.33	d	7.95 ± 0.08	c	7.46 ± 0.06	b	8.43 ± 0.40	d	6.59 ± 0.16	a
Urea mg/dL	12.83 ± 0.29	a	23.67 ± 1.16	d	15.41 ± 1.02	b	15.26 ± 0.44	b	12.00 ± 0.01	a	17.19 ± 0.32	c
ALT U/L	37.25 ± 1.13	f	6.83 ± 0.29	a	8.82 ± 0.16	b	16.06 ± 0.62	c	24.66 ± 0.59	d	28.89 ± 0.19	e
AST U/L	6.84 ± 0.24	a	19.02 ± 0.04	f	12.00 ± 0.01	b	14.11 ± 0.19	c	15.32 ± 0.55	d	17.11 ± 0.20	e
GGT U/L	4.65 ± 0.03	c	3.60 ± 0.23	b	2.39 ± 0.14	a	2.32 ± 0.01	a	2.44 ± 0.25	a	2.45 ± 0.32	a
ALP U/L	27.50 ± 0.40	b	40.94 ± 0.08	d	32.77 ± 0.09	c	10.86 ± 0.08	a	11.00 ± 0.01	a	11.18 ± 0.32	a
Creatinine mg/dL	0.42 ± 0.04	a	0.59 ± 0.02	c	0.50 ± 0.01	b	0.50 ± 0.01	b	0.56 ± 0.11	c	1.04 ± 0.08	d

**Table 2:** Mean ± SD of blood hematological parameters in rats infected by *K. pneumoniae* or *P. aeruginosa* measured at various time points after infection (the same letters denote homogenous groups within a given parameter; ANOVA, Tukey test; *p* < 0.05).

Hematological Parameters	*K. pneumoniae* Infected Rats (Mean ± SD)								*P. aeruginosa* Infected Rats (Mean ± SD)							
	Day 5		Day 10		Day 15		Day 25		Day 5		Day 10		Day 15		Day 25	
WBC	9.52 ± 1.28	ab	11.43 ± 1.17	b	9.07 ± 0.84	ab	10.511.37±	ab	7.61 ± 0.82	a	11.13 ± 0.72	b	9.94 ± 1.08	ab	11.37 ± 1.13	b
RBC	8.89 ± 0.32	a	8.47 ± 1.02	a	8.50 ± 1.15	a	9.40 ± 1.19	a	9.96 ± 0.98	a	8.52 ± 0.92	a	7.71 ± 0.53	a	10.03 ± 1.04	a
HGB	16.06 ± 1.05	a	14.94 ± 1.76	a	14.91 ± 0.65	a	16.84 ± 1.52	a	16.11 ± 1.07	a	14.68 ± 0.59	a	14.35 ± 0.85	a	16.64 ± 0.81	a
HCT	52.54 ± 3.87	a	48.37 ± 5.11	a	48.59 ± 4.77	a	57.86 ± 4.38	a	50.97 ± 4.52	a	46.98 ± 4.09	a	47.88 ± 3.18	a	57.22 ± 5.19	a
MCV	59.91 ± 4.55	a	63.86 ± 3.28	a	65.50 ± 3.88	a	68.11 ± 3.54	a	59.51 ± 3.44	a	63.79 ± 3.91	a	69.07 ± 5.48	a	66.99 ± 4.25	a
MCH	18.87 ± 0.16	a	19.30 ± 1.04	a	19.98 ± 1.33	a	19.70 ± 1.17	a	18.77 ± 1.03	a	19.63 ± 1.12	a	20.37 ± 1.21	a	19.63 ± 1.12	a
MCHC	33.87 ± 1.96	a	32.18 ± 1.52	a	32.31 ± 1.79	a	31.41 ± 1.68	a	33.91 ± 1.67	a	32.69 ± 1.54	a	32.46 ± 1.51	a	31.66 ± 1.43	a
RDW-SD	27.63 ± 1.21	a	31.04 ± 1.92	a	39.82 ± 1.44	b	34.54 ± 2.83	a	26.87 ± 1.91	a	32.32 ± 2.50	a	42.93 ± 1.65	b	42.68 ± 2.62	b
RDW-CV	17.86 ± 1.06	a	17.16 ± 0.82	a	21.05 ± 1.86	a	19.12 ± 1.18	a	18.03 ± 1.31	a	17.63 ± 1.16	a	21.07 ± 2.57	a	22.12 ± 1.47	a
PDW	7.53 ± 0.82	a	7.68 ± 0.82	a	7.50 ± 0.89	a	8.05 ± 1.01	a	8.56 ± 0.91	a	8.50 ± 1.00	a	8.25 ± 0.91	a	8.11 ± 0.89	a
MPV	7.64 ± 0.68	a	7.95 ± 0.59	a	7.56 ± 0.86	a	7.71 ± 0.69	a	7.74 ± 0.72	a	8.33 ± 0.52	a	7.89 ± 0.85	a	7.68 ± 0.83	a
P-LCR	5.72 ± 0.72	a	6.32 ± 0.73	a	4.81 ± 0.52	a	4.91 ± 0.53	a	8.42 ± 0.63	b	8.70 ± 0.82	b	7.13 ± 0.45	b	6.28 ± 0.84	ab
PCT	0.63 ± 0.08	a	0.64 ± 0.02	a	0.79 ± 0.10	a	0.70 ± 0.12	a	0.67 ± 0.08	a	0.76 ± 0.12	a	0.75 ± 0.08	a	0.72 ± 0.06	a
NEUT	14.97 ± 0.59	c	17.62 ± 1.79	d	14.64 ± 1.17	c	7.31 ± 0.09	a	11.03 ± 1.16	b	21.31 ± 2.02	d	17.49 ± 2.07	d	17.32 ± 0.94	d
LYMPH	82.16 ± 2.01	ab	78.19 ± 5.62	ab	81.05 ± 4.83	ab	93.89 ± 6.22	b	81.94 ± 5.92	ab	71.05 ± 4.12	ab	74.88 ± 3.28	ab	66.34 ± 5.37	a
MONO	3.38 ± 0.64	b	5.02 ± 0.86	bc	6.28 ± 0.86	c	1.32 ± 0.23	a	4.83 ± 0.95	b	7.55 ± 1.27	c	4.59 ± 0.72	b	13.77 ± 0.80	d
EO	2.81 ± 0.13	b	2.04 ± 0.51	ab	1.21 ± 0.18	a	1.47 ± 0.30	a	4.87 ± 0.46	c	3.33 ± 0.40	bc	3.84 ± 0.42	c	4.06 ± 0.26	c
BASO	3.74 ± 0.45	a	5.02 ± 0.44	b	4.44 ± 0.33	a	3.91 ± 0.27	a	4.72 ± 0.35	a	4.79 ± 0.51	ab	7.22 ± 0.49	c	6.30 ± 0.52	bc
IG	0.04 ± 0.00	b	0.00 ± 0.00	a	0.00 ± 0.00	a	0.00 ± 0.00	a	0.14 ± 0.03	c	0.42 ± 0.06	d	0.00 ± 0.00	a	0.00 ± 0.00	a

## Data Availability

All data generated and analyzed in this study are included in this article.

## Supplementary Materials

The following supporting information can be downloaded at: https://www.mdpi.com/article/10.3390/pathogens11080933/s1, Figure S1: The % area showing pyknosis and necrosis in the cerebral cortex at different time intervals of infected rats with either (A) *K. pneumoniae*, or (B) *P. aeruginosa* was analyzed; Figure S2: The thickness of the pyramidal layer of the hippocampus at different time intervals of infected rats with either (A) *K. pneumoniae*, or (B) *P. aeruginosa* was assessed.
