# Correction: Elwakil et al. Memory Impairment, Pro-Inflammatory Host Response and Brain Histopathologic Severity in Rats Infected with *K. pneumoniae* or *P. aeruginosa* Meningitis. *Pathogens* 2022, *11*, 933

**DOI:** 10.3390/pathogens13070595

**Published:** 2024-07-17

**Authors:** Bassma H. Elwakil, Basant A. Bakr, Mohammed M. Aljeldah, Nourhan S. Shehata, Yahya H. Shahin, Zakia A. Olama, Maria Augustyniak, Mourad A. M. Aboul-Soud, Abeer El Wakil

**Affiliations:** 1 Department of Medical Laboratory Technology, Faculty of Applied Health Sciences Technology, Pharos University in Alexandria, Alexandria P.O. Box 21311, Egypt; 2 Department of Zoology, Faculty of Science, Alexandria University, Alexandria P.O. Box 21568, Egypt; 3 Department of Clinical Laboratory Sciences, College of Applied Medical Sciences, University of Hafr Al Batin, Hafr Al Batin 39524, Saudi Arabia; 4 Department of Botany and Microbiology, Faculty of Science, Alexandria University, Alexandria P.O. Box 21568, Egypt; 5 Faculty of Natural Sciences, Institute of Biology, Biotechnology and Environmental Protection, University of Silesia in Katowice, Bankowa 9, 40-007 Katowice, Poland; 6 Chair of Medical and Molecular Genetics Research, Department of Clinical Laboratory Sciences, College of Applied Medical Sciences, King Saud University, P.O. Box 10219, Riyadh 11433, Saudi Arabia; 7 Department of Biological and Geological Sciences, Faculty of Education, Alexandria University, Alexandria P.O. Box 21526, Egypt

In the original publication [1], there was a mistake in Figure 5 and Supplementary Figure S2A as published. There was an error in the sub-image of the first row, the fourth column the sub-image (B) in hippocampus day 5 in Figure 5. In Figure S2A, only change the value of granular layer thickness in the hippocampus on day 5. The corrected Figure 5 and Supplementary Figure S2A appear below.

**Figure 5:**
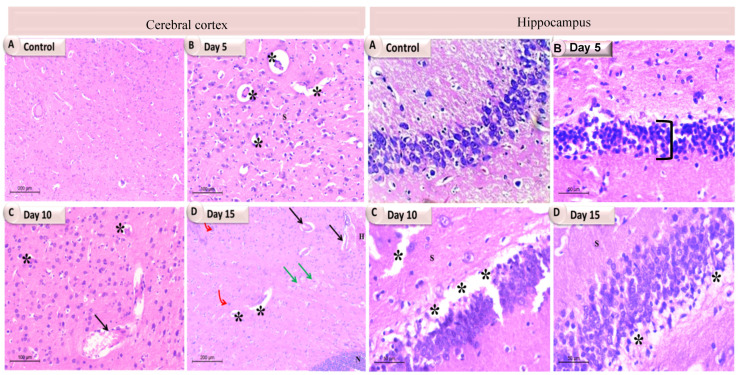
A photomicrograph showing the individual brain tissues at different time intervals of *K. pneumoniae* infected rats. In the cerebral cortex, (**A**) The control group showing normal cytoarchitecture with normal neurons; (**B**) Day 5 showing vacuolations (stars); (**C**) Day 10 showing dilated congested blood vessel (arrow) with perivascular edema and vacuolations (stars); (**D**) Day 15 showing dilated blood vessels (black arrows), severe congestion (green arrows), vacuolations (stars) and gliosis (red bent arrows), a large area of necrotic foci in the brain parenchyma along with lymphocyte infiltration and the presence of degenerating and/or apoptotic neurons (N). While in the hippocampus: (**A**) The control group showing normal cytoarchitecture; (**B**) Day 5 showing decreased thickness of the pyramidal layer (bracket); (**C**) Day 10 showing degeneration and vacuolation (stars); (**D**) Day 15 exhibited a number of vacuolations (stars).

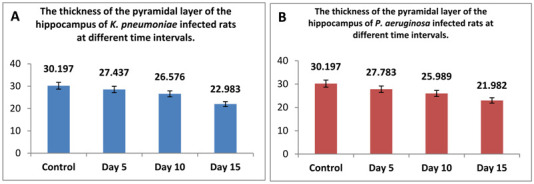



**Supplementary Figure S2:** The thickness of the pyramidal layer of the hippocampus at different time intervals of infected rats with either (**A**) *K. pneumoniae*, or (**B**) *P. aeruginosa* was assessed. Herein, five different fields in each photomicrograph from Figures 5 and 6 at each different time interval of infected rats were analyzed on Intel® Core I7® based computer using VideoTest Morphology® software (Russia) with a specific built-in routine for measuring the thickness of the pyramidal layer of the hippocampus. The difference between *K. pneumoniae* and *P. aeruginosa* in decreasing the thickness of the layer became more evident at day 15 after infection, and it is slightly higher in case of *P. aeruginosa* than *K. pneumoniae*.

The authors state that the scientific conclusions are unaffected. This correction was approved by the Academic Editor. The original publication has also been updated.
