# Supplementary material for: Memory Impairment, Pro-Inflammatory Host Response and Brain Histopathologic Severity in Rats Infected with K. pneumoniae or P. aeruginosa Meningitis
Source: Pathogens. 2022 Aug 18;11(8):933. doi: 10.3390/pathogens11080933 (PMC9416464; doi:10.3390/pathogens11080933)
Supplement: Supplementary file 1 [file pathogens-11-00933-s001.zip › pathogens-1833681-supplementary.pdf]

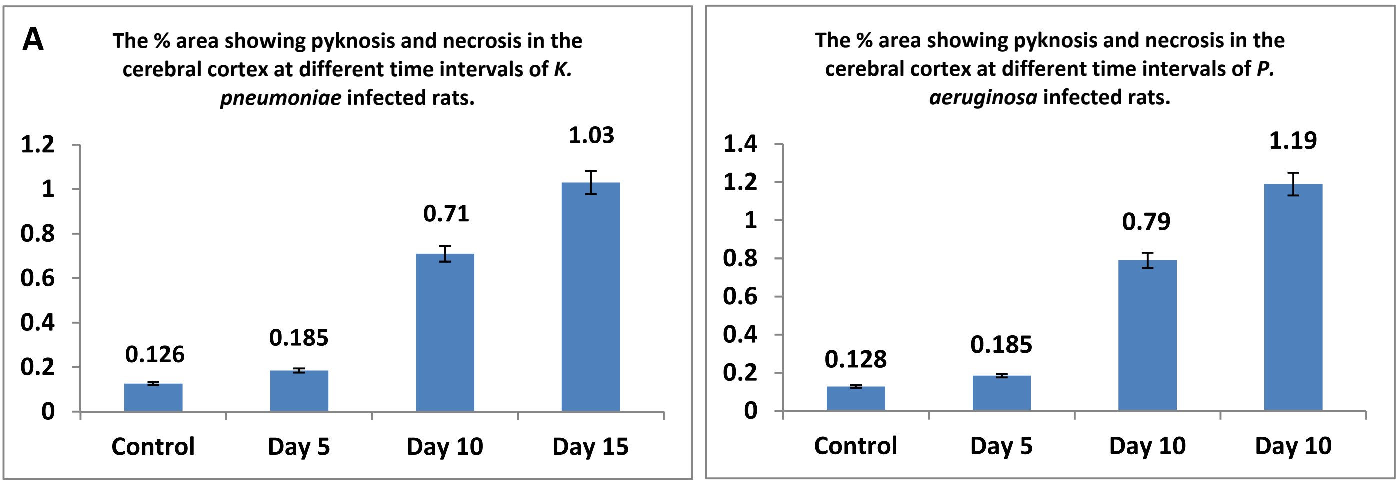

Supplementary Figure S1: The % area showing pyknosis and necrosis in the cerebral cortex at different time intervals of infected rats with either (**A**) *K. pneumoniae*, or (**B**) *P. aeruginosa* was analyzed. Herein, five different fields in each photomicrograph from Figures 5 & 6 at each different time interval of infected rats were analyzed on Intel® Core I7® based computer using VideoTest Morphology® software (Russia) with a specific built-in routine for area, % area, measurement, and object counting. There is no significant difference between both bacterial infection on inducing pyknosis and necrosis in the cerebral cortex at different time intervals of infected rats. However, the longer the time of infection, the more observed pyknotic and necrotic cells particularly with *P. aeruginosa*.

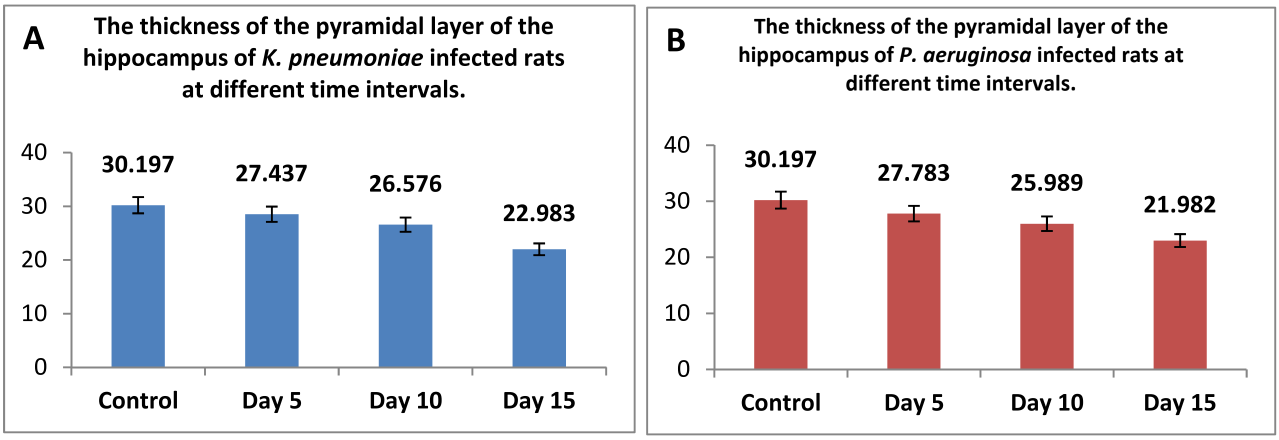

Supplementary Figure S2: The thickness of the pyramidal layer of the hippocampus at different time intervals of infected rats with either (**A**) *K. pneumoniae,* or (**B**) *P. aeruginosa* was assessed. Herein, five different fields in each photomicrograph from Figures 5 and 6 at each different time interval of infected rats were analyzed on Intel® Core I7® based computer using VideoTest Morphology® software (Russia) with a specific built-in routine for measuring the thickness of the pyramidal layer of the hippocampus. The difference between *K. pneumoniae* and *P. aeruginosa* in decreasing the thickness of the layer became more evident at day 15 after infection, and it is slightly higher in case of *P. aeruginosa* than *K. pneumoniae.*
